# Diagnostic test accuracy of teleretinal screening for cytomegalovirus retinitis among people living with HIV: A systematic review and meta-analysis

**DOI:** 10.1371/journal.pgph.0006327

**Published:** 2026-05-22

**Authors:** Holijah Uy, Leandrea Nollora, Matthew Villanueva, John Mark De Leon, Nilo D. G. FlorCruz, Albert John Bromeo

**Affiliations:** Department of Health Eye Center, East Avenue Medical Center, Quezon City, Philippines; University of Colorado Anschutz Medical Campus: University of Colorado - Anschutz Medical Campus, UNITED STATES OF AMERICA

## Abstract

Cytomegalovirus retinitis (CMVR) remains a major cause of preventable blindness among people living with human immunodeficiency virus (PLHIV), particularly in resource-limited settings where access to ophthalmologists is constrained. Teleretinal screening offers a practical strategy for early detection and timely referral, with the potential to prevent irreversible visual impairment. This review aimed to determine the diagnostic accuracy of teleretinal screening for CMVR in PLHIV. It was conducted in accordance with the PRISMA diagnostic test accuracy guidelines and was registered prospectively in PROSPERO (CRD420250637110). The Cochrane Library, PubMed, CINAHL, Scopus, and Web of Science were searched on March 1, 2025. Risk of bias was assessed using the QUADAS-2 tool. Sensitivity and specificity values were pooled using the hierarchical summary receiver operating characteristic (HSROC) model, and heterogeneity was visually examined using SROC and forest plots. Subgroup analyses were performed according to country income setting, CD4 cell count thresholds, and fundus imaging modality. Quality of evidence was assessed using the GRADE framework. Five studies involving 1,460 eyes of PLHIV were included. Meta-analysis demonstrated a pooled sensitivity of 87.11% (95% confidence interval [CI]: 50.35–100%; low-certainty evidence) and a specificity of 97.73% (95% CI: 89.88–100%; high-certainty evidence). Subgroup analyses suggested higher sensitivity among populations with lower CD4 cell count thresholds. Sensitivity analysis showed no significant changes after excluding studies with a high risk of bias. Teleretinal screening for CMVR demonstrates consistently high specificity and potentially useful sensitivity. supporting its role in early detection and referral, particularly among PLHIV with advanced HIV disease. By integrating retinal imaging at the point of care with remote interpretation by ophthalmologists, teleretinal screening can expand access to eye care and reduce diagnostic delays in underserved settings. Nonetheless, further high-quality studies in diverse lower-resource settings are needed to strengthen the evidence base and guide scalable implementation within HIV care systems.

## Introduction

Communicable diseases lag significantly behind the leading global causes of avoidable blindness [1]. The current definition of vision impairment sets a visual acuity threshold based on the better-seeing eye, overlooking the impact of conditions that affect only one eye, which is more prevalent in communicable diseases [1]. In addition, many epidemiologic studies on global trends focused on a population >50 years old, as their data were based on Rapid Assessment of Avoidable Blindness (RAAB) studies, which focused on people >50 years of age [2]; thus, not representing many essential causes of blindness, such as childhood blindness and infectious ocular diseases that are more prevalent in younger populations. Moreover, most sight-threatening communicable diseases are underreported, especially in low- and middle-income countries (LMICs), due to a lack of registry and scarcity of diagnostic and screening services [3–5].

Cytomegalovirus retinitis (CMVR) is one of the neglected sight-threatening ocular diseases caused by the communicable disease, CMV infection [6,7]. It is the most common opportunistic ocular manifestation of systemic CMV infection occurring in immunocompromised individuals, particularly those with advanced HIV infection [3]. With the advent of highly active antiretroviral therapy (HAART), the incidence of CMVR has decreased significantly by 80% in developed countries [8]. However, in many developing countries, including Southeast Asia, the prevalence of CMVR remains concerningly high and remains a significant sight-threatening disease in countries with a high burden of HIV [9].

A systematic review investigating the prevalence of CMVR in LMICs found that Asia has the highest prevalence (14%), followed by Latin America (12%) and Africa (2%) [10]. Among Asian countries, CMVR has a prevalence of 24.8% in Myanmar, 24.4% in Thailand, 15.2% in China, and 6.8% in India [10]. This pattern parallels the continued burden of HIV disease in these regions, where a substantial proportion of people living with HIV (PLHIV) present severe immunosuppression, thereby contributing to the persistently high prevalence of CMVR [11].

Management of CMVR is primarily medical, involving intravenous, oral, and intravitreal antiviral medications [5]. In PLHIV, treatment is also dependent on HAART. Patients with CMVR should begin with high-dose induction therapy, followed by ongoing maintenance therapy until CD4 cell counts rise, HAART becomes therapeutic, and CMVR shows no progression [12]. However, it is important to note that PLHIV with CMVR receiving HAART can develop immune reconstitution inflammatory syndromes (IRIS) such as immune recovery uveitis (IRU) and immune recovery retinitis (IRR) as the immune system recovers [13]. The most serious IRIS with CMVR is IRU. This usually leads to blurred vision, cystoid macular edema, epiretinal membrane formation, posterior capsular cataracts, proliferative vitreoretinopathy, and optic nerve neovascularization [12]. Thus, aside from screening PLHIV with CMVR for timely treatment and management, screening newly diagnosed PLHIV before initiating HAART is strongly recommended to prevent these complications.

Cytomegalovirus retinitis remains the neglected disease of the HIV pandemic, with an increasing number of young patients in the LMICs whose health has improved due to effective HAART treatment but are left permanently blind because of undiagnosed or under-treated CMVR [14]. In some LMICs with limited resources, screening and management of CMVR are inadequate [3,15,16]. As a result, patients are often referred to ophthalmologists once CMVR has progressed to an advanced stage, leading to poor outcomes [4].

At present, there is no clear consensus on who should undergo screening, the optimal screening interval, or the most appropriate methods [5]. Although routine retinal examinations are advised for high-risk populations, particularly individuals with HIV and CD4 counts below 50 cells/mm^3^, practical implementation remains challenging due to the limited availability of resources and trained personnel [5]**.** Several screening methods have been explored for CMVR in settings with limited access to ophthalmic resources. One method is teleretinal screening, which utilizes a fundus camera to capture fundus images at the primary care level and transmit them to partner institutions with trained ophthalmologists for reading and interpretation [3,16].

Currently, a limited number of published studies have evaluated the diagnostic accuracy of teleretinal screening for CMVR among PLHIV. However, there are still no systematic reviews or meta-analyses that report pooled sensitivity and specificity for teleretinal screening of the target disease. Therefore, we conducted a systematic review and meta-analysis to provide evidence-based recommendations for integrating teleretinal screening for CMVR, particularly in settings with limited access to ophthalmologists.

## Methods

### Protocol‌‌

We conducted this review in accordance with the Preferred Reporting Items for Systematic Reviews and Meta-Analyses of Diagnostic Test Accuracy Studies (PRISMA-DTA) guidelines [17]. The study protocol was registered prospectively with the International Prospective Register of Systematic Reviews (PROSPERO) under the identifier CRD420250637110. An ethics waiver was granted by the East Avenue Medical Center Institutional Ethics Review Board.

### Eligibility criteria

#### Type of studies.

We included prospective studies evaluating the DTA of teleretinal screening in this review. We excluded retrospective studies using already available images. We also excluded review articles, case reports, and qualitative studies.

#### Type of participants.

We included PLHIV regardless of age, sex, CD4 count, race, and geographical location.

#### Setting.

We included studies conducted in primary, secondary, and tertiary healthcare settings.

#### Index test.

We included interventions that used teleretinal screening for CMVR, in which fundus images were captured with a fundus camera and graded by ophthalmologists.

#### Reference standard.

We included reference standards that used a dilated fundus exam (DFE) conducted by ophthalmologists using an indirect ophthalmoscope. We excluded reference standards that used grading of fundus images by ophthalmologists rather than DFE in person.

#### Target condition.

We included studies that screened for CMVR, regardless of zone or pattern. Eyes deemed ungradable or inconclusive during DFE by ophthalmologists were excluded from the pooling of diagnostic accuracy outcomes.

### Outcome measures

We included studies that reported, or from which it was possible to derive, the data needed to construct contingency tables with the following proportions: true positives (TP), false positives (FP), true negatives (TN), and false negatives (FN). When relevant data were missing or not presented in the main text or supplementary materials, corresponding authors were contacted to obtain the necessary information.

### Search methods

We searched the following electronic databases on March 1, 2025, without any language or year restrictions: Cochrane Central Register of Controlled Trials (CENTRAL), Medical Literature Analysis and Retrieval System Online (MEDLINE) via PubMed, Cumulative Index to Nursing and Allied Health Literature (CINAHL), Scopus, and Web of Science. We also hand-searched the reference lists of relevant primary studies.

### Study selection

We used Rayyan software to manage the retrieved studies. Review authors [HU and LN] independently screened the titles and abstracts and classified them as (a) included, (b) maybe, and (c) excluded. Full-text articles of those ‘included’ and ‘maybe’ were obtained and independently assessed by the same authors against the eligibility criteria. Studies were then classified as (a) included, (b) excluded. Any disagreements between the two reviewers were resolved through discussion or consultation with a third review author [AB].

### Data extraction and management

Using Excel, we extracted the study characteristics and developed a data extraction form to compute the study outcomes: TP, FP, FN, TN. Data were extracted by two review authors [HU and MV] between April and June 2025.

### Risk of bias and applicability

The risk of bias and applicability on the (a) patient selection, (b) index test, (c) reference standard, and (d) flow and timing of the included studies were independently assessed by two review authors [HU and MV] using the Quality Assessment of Diagnostic Accuracy Studies (QUADAS)-2 tool [18]. Any disagreements between the two authors were resolved through discussion or consultation with a third senior review author [AB].

### Data synthesis and analysis

We calculated the sensitivity and specificity of the included studies at the eye-level analysis. Since this is a DTA systematic review, heterogeneity was explored using visual inspection of forest plots and hierarchical summary receiver operating characteristics (HSROC) plots. The analyses and plots were generated using SAS Studio and Review Manager 5.4 (RevMan).

#### Subgroup analysis.

We performed subgroup analyses on the following covariates: level of economic development (World Bank country classification), CD4 cell count threshold, and fundus imaging modality. However, we were unable to conduct subgroup analyses by study setting, as all studies were conducted in tertiary healthcare settings, with one study also conducted in both primary and tertiary healthcare settings.

#### Sensitivity analysis.

We performed a sensitivity analysis to assess the impact of excluding a study with a high risk of bias in the patient domain, as assessed by the QUADAS-2 tool.

### Certainty of the body of evidence and summary of findings

We applied the summary estimates to a hypothetical cohort of 100 patients in our overall analysis using the Grading of Recommendations, Assessment, Development and Evaluation (GRADE)pro guideline development tool by McMaster University.

## Results

### Characteristics of included studies

**Fig 1:**
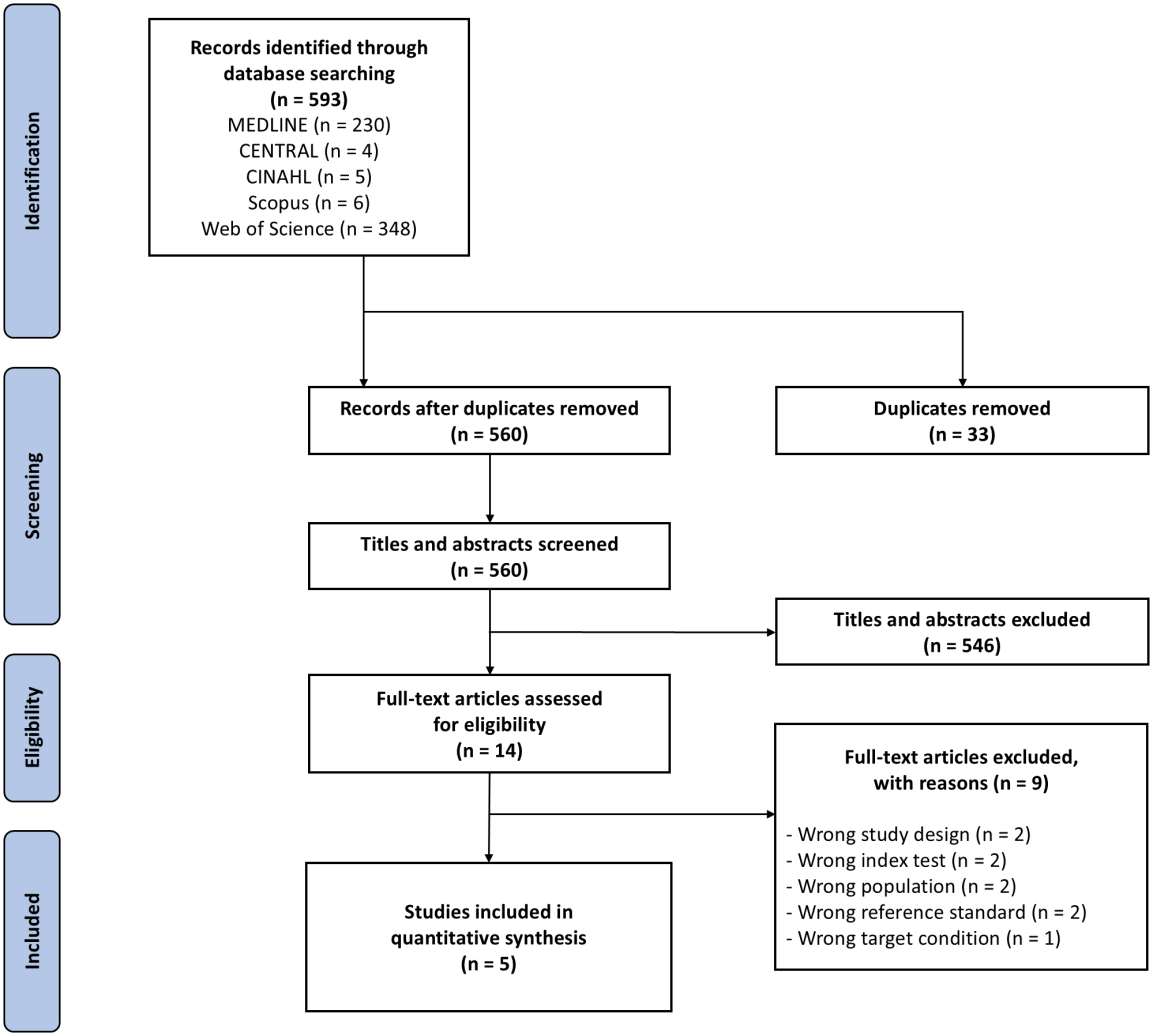
PRISMA flow diagram of the study search and selection.

We identified 593 articles from the following databases: CENTRAL, MEDLINE via PubMed, CINAHL, Scopus, and Web of Science. After deduplication, 560 articles were screened based on titles and abstracts, of which 546 were excluded. The remaining 14 articles were screened for full-text assessment against the review’s eligibility criteria, finally leaving five studies for quantitative synthesis (**Fig 1**).

**Table 1** shows the characteristics of the five included studies published between 2011 and 2022 (11-year span). All studies were done prospectively. For the study setting, three studies were conducted in Thailand, and one in China, all of which are classified as upper-middle-income countries (U-MICs) under the broader LMIC category, and one study was conducted in Singapore, a high-income country (HIC). All studies were conducted in a tertiary-level healthcare setting, while one study was conducted in both primary- and tertiary-level healthcare settings. Two studies were conducted in the hospital’s HIV clinics, and one was conducted in an ocular infectious disease clinic. For patient characteristics, two studies set a CD4 cell count threshold of <100 cells/mm^3^, and one study set thresholds of <200 and <50 cells/mm^3^; the other study did not mention a CD4 cell count threshold. Two studies utilized the Topcon TRC-NW 6S digital fundus camera, which processed composites of nine images covering 85 degrees of the retinal field. Another study employed a Zeiss FF450 fundus camera with a Kodak DCS620 digital back, which also processed composites of nine images covering 95–105 degrees of the retinal field. One study used an Optos ultra-widefield fundus camera that captured a single image covering 200 degrees of the retinal field. Another study employed a KOWA nonmyd alphaD III/VK-2 digital imaging system, which captured a single-center fundus image covering 45 degrees of the retinal field. In all studies, fundus photos were captured by trained personnel, teleretinal screenings were read by ophthalmologists, and DFEs were performed by ophthalmologists.

**Table 1:** Key characteristics of the study settings, population, index test, and reference standard of included studies.

Study	Study Settings			Patient Characteristics			Index Test				Reference Standard
	Country (WBC)	Setting	Healthcare Setting	Age, mean (SD), years	CD4 Cell Count Threshold, cells/mm^3^	Sample Size, Eyes ^a^	Fundus Camera Used	Fundus Images (Retinal Field Covered)	Fundus Photo Captured by	Teleretinal Screening Performed by (№)	Dilated Fundus Exam Done by
**Ausayakhun 2011** [19]	Thailand (U-MIC)	Hospital (Ocular Infectious Disease Clinic)	Tertiary	38.67 (±8.89)	<100	182	Topcon TRC-NW 6S digital fundus camera	Composite of 9 images (85^◦^)	Trained photographer	Uveitis specialists (3)	Ophthalmologist
**Du 2020** [20]	China (U-MIC)	Hospital	Tertiary	38.31 (±10.53)	<200	186	Optos Ultra-widefield	Single image (200^◦^)	Ophthalmologist	Ophthalmologist	Ophthalmologist
**Jirawison 2015** [21]	Thailand (U-MIC)	Hospital (HIV Clinic)	Tertiary	36.67 (±10.37)	<100	205	Topcon TRC-NW 6S digital fundus camera	Composite of 9 images (85^◦^)	Trained photographer	Retina specialists (3)	Retina specialist
**Shah 2013** [22]	Singapore (HIC)	Hospital	Tertiary	43.90 (±11.0)	<50	724	Zeiss FF450 fundus camera with a Kodak DCS620 digital-back	Composite of 9 images (95^◦^-105^◦^)	Qualified technician	Retina specialists	Retina specialist
**Srisuriyajan 2022** [23]	Thailand (U-MIC)	Hospital (HIV Clinic)	Primary and Tertiary	42.00 (±8.17)	*Not mentioned*	163	KOWA nonmyd alphaD III/VK-2 digital imaging system	Single, center fundus image (45^◦^)	Trained photographer	Third-year ophthalmology resident	Retina specialist

^a^Sample included in the diagnostic accuracy analysis excluding images deemed ungradable by the reference standard.

**HIC**, high-income country; **SD**, standard deviation; **U-MIC**, upper middle-income country; **WBC**, World Bank classification.

### Risk of bias assessment

**Fig 2:**
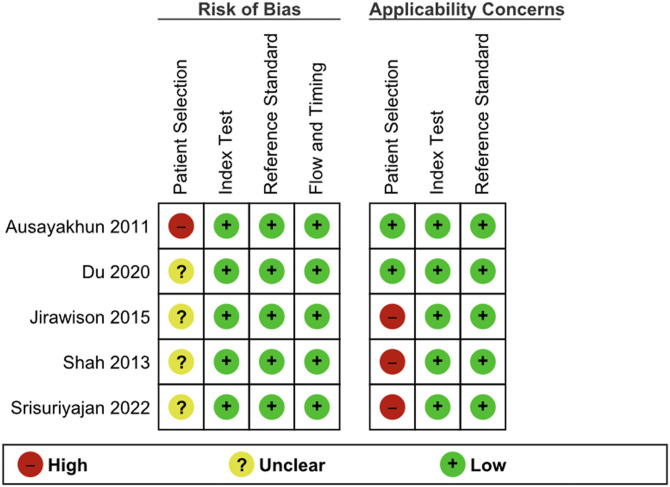
Risk of bias and applicability concerns summary: review authors' judgments about each domain for each included study using the QUADAS-2 tool.

**Fig 3:**
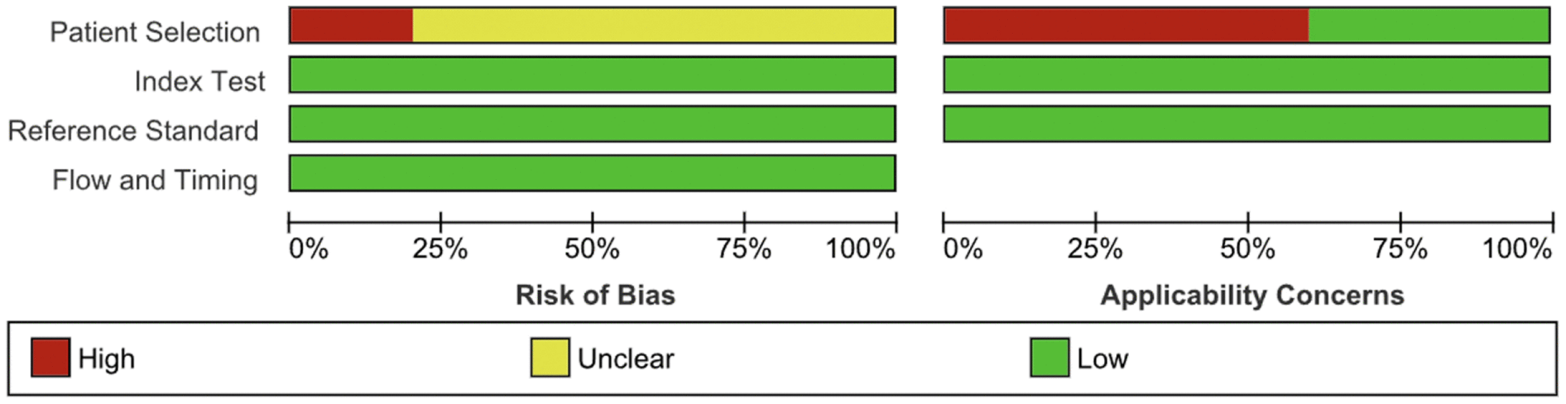
Risk of bias and applicability concerns graph: review authors' judgments for each domain presented as percentages across included studies using the QUADAS-2 tool.

Summaries of methodological quality assessment using the QUADAS-2 tool were presented in **Figs 2 and 3**.

#### Patient selection.

In the patient selection domain, all studies were deemed to have an unclear risk of bias due to the signaling question about the sampling method and patient enrollment. No studies used a case-control study design. We deemed the study by Ausayakhun et al. (2011) to have a high risk of bias, as it included patients with previously diagnosed CMVR, which may have exaggerated the diagnostic accuracy [19]. Three studies were deemed to have a high level of concern regarding the applicability of patent selection. Both studies by Jirawison et al. (2015) and Srisuriyajan et al. (2022) did not include patients under 18 years of age, despite the prevalence of HIV rising in this age group [21,23]. The study by Shah et al. (2013) was also judged to have a high concern for applicability because PLHIV previously diagnosed with CMVR with inactive, current residual lesions were not considered to have CMVR, whereas in the real-world setting, active or inactive CMVR should be screened and referred to the ophthalmology service for monitoring [22].

#### Index test.

In the domain of the index tests, all studies were deemed to have low risk of bias. The quality of images was ensured during the teleretinal screening process in all studies. Reading and interpreting the images were done without knowledge of the DFE results. No thresholds were set for teleretinal screening, particularly when using fundus cameras to capture the retina. All studies were deemed to have low concern for applicability.

#### Reference standard.

In the domain of reference standard, all studies were deemed to have a low risk of bias. Dilated fundus examination by an ophthalmologist is the gold standard for clinically identifying CMVR in all settings. In all studies, ophthalmologists were blinded to the results of the teleretinal screening. All studies were deemed to have low concern for applicability as well.

#### Flow and timing.

In the flow and timing domain, all studies were deemed to have a low risk of bias. There was an appropriate interval between the time the fundus photos were taken and when they were interpreted via DFE in all studies. All eyes were included in the study except those deemed ungradable during the DFE. Only evaluable eyes, as determined by DFE, were included to reduce the risk of misclassification caused by poor-quality images captured during teleretinal screening. This domain is not assessed regarding applicability concerns, as stated in the QUADAS-2 tool.

### Outcomes

We evaluated the accuracy of teleretinal screening for CMVR compared with standard DFE performed by ophthalmologists. All five studies were evaluated via eye-level analysis rather than patient-level analysis. Eye-level analysis aligns better with real-world clinical practice, where the condition of CMVR and its progression are assessed per eye. Also, if warranted, intravitreal ganciclovir injections are administered per eye, especially in resource-poor settings [3].

In DTA meta-analyses, heterogeneity is commonly assessed by visual inspection of forest plots and HSROC curves, as conventional measures such as I^2^ are not appropriate given the paired and threshold-dependent nature of sensitivity and specificity.

The HSROC model was employed in the meta-analysis for this review, as teleretinal screening processes exhibit variability in the threshold between studies and are not fixed across studies. This model accounts for both within-study and between-study variability, resulting in a more comprehensive summary of test performance. We performed subgroup analyses based on the level of economic development (LMICs vs. HICs), CD4 cell count threshold, and fundus imaging modality (FIM) used.

#### Overall analysis.

**Fig 4:**
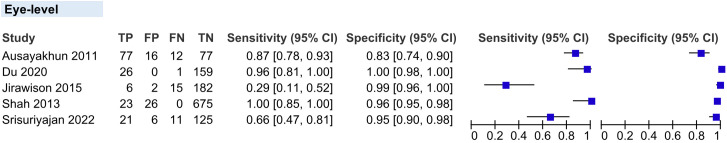
Coupled forest plot of included studies for overall analysis.

**Fig 5:**
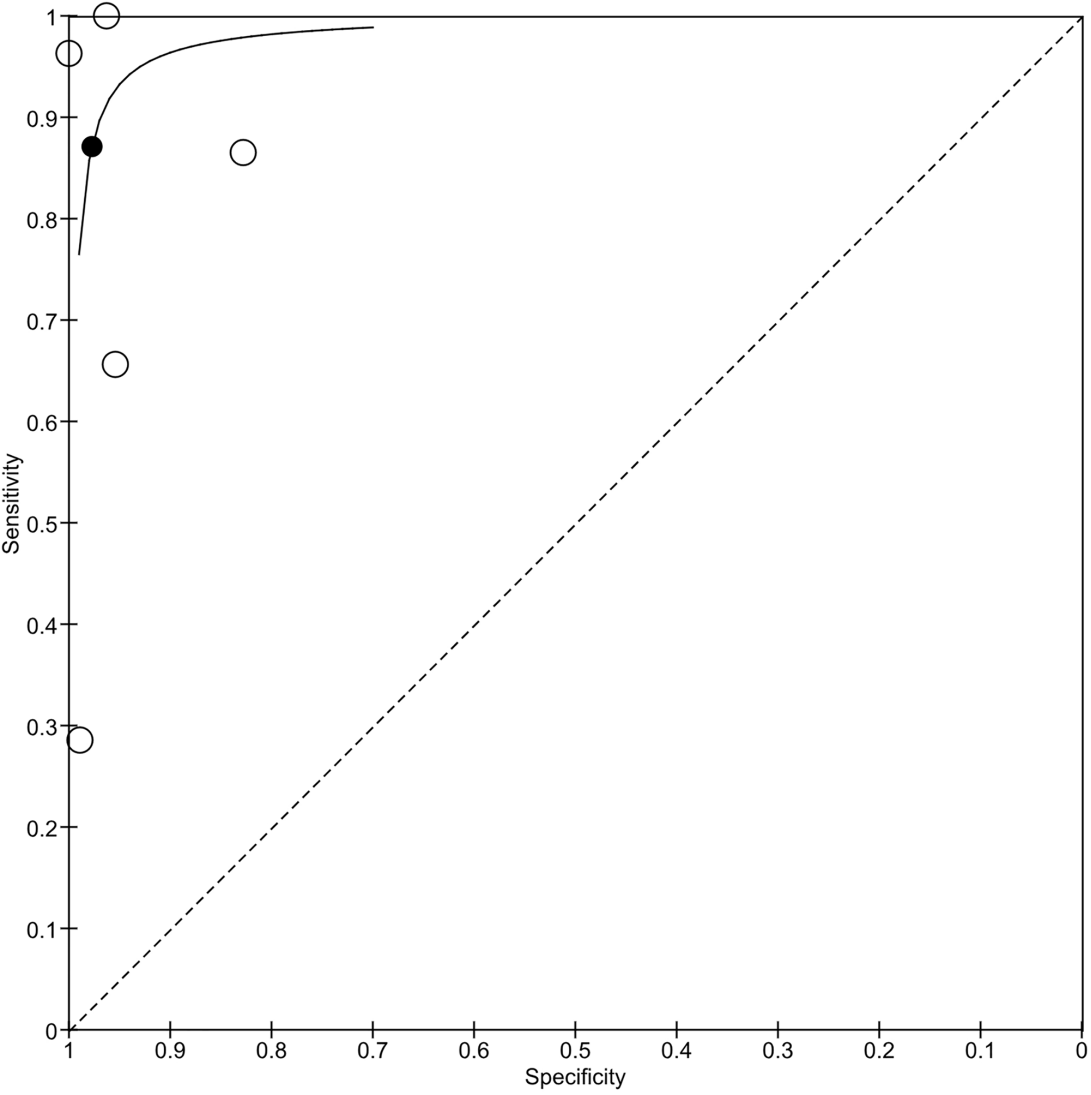
HSROC plot of sensitivity vs specificity of teleretinal screening for detecting CMVR.

Five evaluations of teleretinal screening for CMVR were conducted across five studies, involving 1460 eyes. The forest plot shows substantial variation in sensitivity and moderate variation in specificity (**Fig 4**). The HSROC plot (**Fig 5**) exhibits an upper left asymptotic shape, indicating high specificity but variable sensitivity across studies. The summary point (black dot) represents the pooled sensitivity and specificity, which are approximately 0.87 and 0.97, respectively. The spread of circles indicates high variability in sensitivity and low variability in specificity. The meta-analytical sensitivity and specificity were 87.11% (95% CI: 50.35-100%) and 97.73% (95% CI: 89.88-100%), respectively.

#### Exploring heterogeneity.

We performed subgroup analyses to explore potential sources of heterogeneity. We performed subgroup analyses by level of economic development, CD4 cell count threshold, and fundus imaging modality. Four studies were conducted solely in tertiary-level healthcare settings, and only one was conducted in a primary-level healthcare setting, but the data were combined with those from a tertiary-level setting. Detailed results of subgroup analyses investigating potential sources of study-level heterogeneity are shown in **Table 2**.

**Table 2:** Subgroup analyses for the accuracy of teleretinal screening‌‌ in detecting CMVR compared with dilated fundus examination.

Analysis		№ of Studies	№ of Eyes	Sensitivity (95% CI)	Specificity (95% CI)
**Overall Meta-analysis**					
Eye-level		5	1460	87.11% (50.35-100)	97.73% (89.88-100)
**Subgroup Analyses**					
Level of economic development	LMIC	4	736	76.42% (49.24-100)	97.93% (93.84-100)
	HIC	1	724	100% (85.00-100)	96.29% (94.89-97.69)
CD4 count threshold (cells/mm^3^)	<200	1	186	96.30% (81.00-100)	100% (98.00-100)
	<100	2	387	62.01% (14.99-100)	95.36% (85.89-100)
	<50	1	724	100% (85.00-100)	96.29% (94.89-97.69)
Fundus imaging modality	SFI	1	163	65.62% (49.15-82.10)	95.42% (91.84-99.00)
	MFI	3	1111	84.45% (48.49-100)	95.73% (89.96-100)
	UWF	1	186	96.30% (81.00-100)	100% (98.00-100)

Data calculated using SAS Studio.

**CD4**, cluster of differentiation; **HIC**, high-income country; **LMIC**, low- and middle-income country; **MFI**, multi-field composite imaging; **SFI**, single-field imaging; **UWF**, ultra-widefield imaging.

#### Level of economic development.

**Fig 6:**
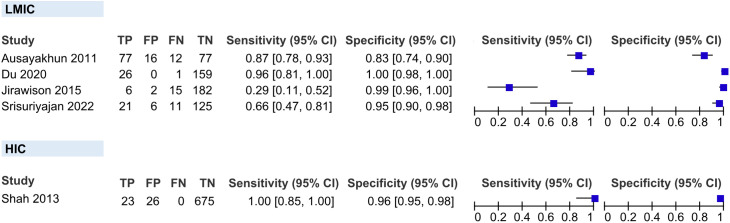
Coupled forest plots showing the subgroups in the level of economic development according to the World Bank country classification.

We classified the countries’ level of economic development using the World Bank Group [24]. All studies included were conducted in Asia. Four studies were conducted in LMICs (Thailand and China), and one in an HIC (Singapore). The pooled sensitivity and specificity of teleretinal screening for CMVR in PLHIV in LMIC were 76.42% (95% CI 49.24-100%) and 97.93% (95% CI 93.84-100%), respectively. In HIC, the study by Shah et al. (2013) has a sensitivity of 100% (95% CI 85–100%) and 96.29% (95% CI 94.89-97.69%), respectively (**Fig 6)** [22].

#### CD4 cell count threshold.

**Fig 7:**
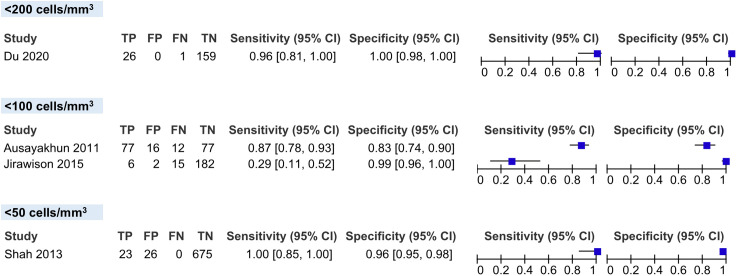
Coupled forest plots showing the subgroups in the CD4 cell count threshold.

We classified the CD4 cell count threshold into three categories: less than 200 cells/mm^3^, less than 100 cells/mm^3^ and less than 50 cells/mm^3^. We were able to pool the sensitivity and specificity of two studies on CD4 cell count thresholds below 100 cells/mm3: 62.01% (95% CI 14.99-100%) and 95.36% (95% CI 85.89-100%), respectively. For the CD4 cell count threshold of <200 cells/mm^3^, the sensitivity and specificity were 96.30% (95% CI, 81.00-100%) and 100% (95% CI, 98.00-100%), respectively. For the CD4 cell count threshold of less than 50 cells/mm^3^, the sensitivity and specificity were 100% (95% CI, 85.00-100%) and 96.29% (95% CI, 94.89-97.69%), respectively **(Fig 7**).

#### Fundus imaging modality.

**Fig 8:**
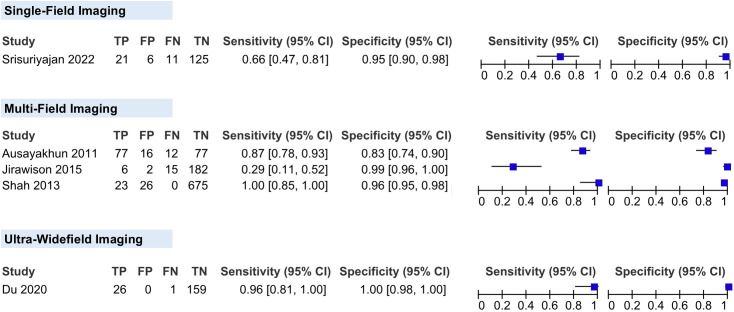
Coupled forest plots showing the subgroups of the fundus imaging modality.

We classified the fundus imaging modality into the following categories: single-field imaging, multi-field imaging (composite of nine images), and ultra-widefield imaging. For multi-field imaging, we pooled three studies in this category, yielding a pooled sensitivity of 84.45% (95% CI, 48.49-100%) and a specificity of 95.73% (95% CI, 89.96-100%). For single-field imaging, the sensitivity and specificity were 65.62% (95% CI, 49.15-82.10) and 95.42% (95% CI, 91.84-99.00), respectively. For the ultra-widefield imaging, the sensitivity and specificity were 96.30% (95% CI 81.00-100%) and 100% (95% CI 98.00-100%), respectively (**Fig 8**).

### Sensitivity analysis

We performed sensitivity analyses by removing studies with a high risk of bias in QUADAS‐2 domains. For this review, we identified one study with a high risk of bias in the patient selection domain. We performed a sensitivity analysis, yielding a pooled sensitivity of 87.71% (95% CI, 26.39-100%) and a pooled specificity of 98.15% (95% CI, 93.85-100%). The exclusion of this study showed the same sensitivity and specificity of teleretinal screening for CMVR in PLHIV **(Table 3**).

**Table 3:** Sensitivity analysis for the accuracy of teleretinal screening for CMVR in PLHIV.

Analysis	№ of Studies	№ of Eyes	Sensitivity (95% CI)	Specificity (95% CI)
**Overall Meta-analysis**				
Eye-level	5	1460	87.11% (50.35-100)	97.73% (89.88-100)
**Sensitivity Analyses**				
After omitting the study with a high risk of bias	4	1278	87.71% (26.39-100)	98.15% (93.85-100)

#### Investigation of publication bias.

A quantitative evaluation of reporting bias was not performed because the most effective method for assessing reporting bias in DTA studies remains under debate, and conventional approaches such as funnel plots are not directly applicable due to the paired and threshold-dependent nature of sensitivity and specificity [25]. In addition, given the small number of included studies, alternative methods such as Deeks’ funnel plot would be underpowered and potentially misleading [25].

### Discussion summary of main findings

This systematic review and meta-analysis evaluated the DTA of teleretinal screening for CMVR among PLHIV, incorporating various fundus camera types across different settings, with varying CD4 cell counts. Our findings suggest that teleretinal screening demonstrates consistently high specificity and potentially useful sensitivity; however, the wide confidence intervals for sensitivity indicate substantial imprecision and uncertainty. Therefore, while the results are promising, they should be interpreted with caution. We found lower sensitivity and specificity in the teleretinal screening in LMICs than in HICs. This discrepancy may reflect differences in image acquisition quality, grader expertise, availability of confirmatory diagnostic tools, and infrastructure for teleretinal screening implementation. Caution should be taken when interpreting this finding, as only one study was available to represent HIC.

Although our review showed that teleretinal screening for CMVR demonstrates higher sensitivity when performed among PLHIV with CD4 counts below 50 cells/mm^3^ compared with thresholds of <100 or <200 cells/mm^3^, this pattern is expected. Retinal lesions, including CMVR, become markedly more prevalent and more clinically apparent as immunosuppression worsens [26]. Studies consistently show that CMVR occur at substantially lower CD4 levels; therefore, the improved diagnostic performance at the < 50 cells/mm^3^ threshold likely reflects the higher disease burden and more obvious retinal changes in this severely immunocompromised subgroup, rather than superior screening technology alone. Based on the available evidence, we recommend a targeted screening approach for CMVR among PLHIV with advanced immunosuppression, particularly those with a CD4 count <100 cells/mm^3^, with the highest priority given to those with CD4 counts <50 cells/mm^3^, where disease burden and detectability are greatest. In addition, the mean age of participants across included studies ranged from 38 to 42 years, suggesting that screening may be particularly relevant among adult PLHIV. However, given the limited representation of adolescents and younger populations, current evidence is insufficient to support age-specific screening thresholds. Therefore, screening strategies should primarily be guided by immunologic status rather than age, while future research should further evaluate the role of age in optimizing screening protocols.

Teleretinal screening using an ultra-widefield system has the highest diagnostic accuracy, followed by multifield imaging with a composite of nine images and single-field imaging. The sensitivity and specificity are also high with MFI with composite imaging. Therefore, in the absence of an ultra-widefield system, composite imaging can be used for screening. We also explored the effect of excluding studies with a high risk of bias and found no significant variation in diagnostic accuracy.

We applied the summary estimates to a hypothetical cohort of 100 patients in our overall analysis using the Grading of Recommendations, Assessment, Development and Evaluation (GRADE)pro guideline development tool by McMaster University (**Table 4**) [27]. Our findings indicate that integrating teleretinal screening for CMVR into the healthcare system will result in 87% of patients with CMVR being correctly identified as having the condition, and 98% of patients without CMVR being correctly identified as not having the condition. This review aimed to determine how many patients would be accurately referred and how many would be needlessly directed to tertiary care for further eye evaluation. The prevalence of CMVR in Asia was 14%, which was used for the GRADE framework, encompassing the countries included in our study [10]. Given this prevalence, teleretinal screening will correctly detect CMVR in 12 PLHIV, but it will miss two CMVR cases and unnecessarily refer two PLHIV cases without CMVR. Although teleretinal screening may occasionally identify other necrotizing herpetic retinitides such as acute retinal necrosis (ARN) or progressive outer retinal necrosis (PORN), these cases would still warrant referral, as the screening pathway emphasizes early detection and management of any potentially sight-threatening retinitis rather than strict etiologic differentiation at the screening level.

**Table 4:** Summary of findings of the review evaluated using the GRADE framework.

Review question: What is the diagnostic test accuracy of teleretinal screening for CMVR compared with DFE by ophthalmologists among PLHIV?				
Population: PLHIV				
Setting: Tertiary-level healthcare settings				
Index test: Teleretinal screening using a fundus camera with retinal images graded by ophthalmologists				
Reference standard: DFE by ophthalmologists				
Study design: Cross-sectional studies with prospective data collection				
Total № of studies: 5 studies (1460 eyes)				
Effect (95% CI)	Test Result	№ of results per 100 Eyes Tested (95% CI)	№ of Eyes (Studies)	Certainty of the Evidence (GRADE)
		Prevalence of 14% ^a^		
**Eye-level analysis**				
**Pooled sensitivity** 87% (50-100%)	**True Positive**	**12** (7-14)	192 eyes (5 studies)	⊕⊕◯◯ **LOW** ^b, c, d^
	**False Negative**	**2** (0-7)		
**Pooled specificity** 98% (90-100%)	**True Negative**	**84** (77-86)	1268 eyes (5 studies)	⊕⊕⊕⊕ **HIGH** ^b^
	**False Positive**	**2** (0-9)		

^a^Prevalence of CMVR in Asia [10].

^b^**Risk of bias:** The QUADAS-2 tool was used to assess the risk of bias. In the Patient Selection domain, the risk of bias was high in one study. However, after conducting a sensitivity analysis, the pooled specificity remained unchanged. Thus, we did not downgrade this effect on this aspect.

^c^**Inconsistency (-1):** Statistical heterogeneity based on the forest plot showed substantial variation in sensitivity.

^d^**Imprecision (-1):** The CI of the pooled sensitivity is wide, indicating uncertainty in the estimate and that the actual value could be lower.

**Grade Definition** [27].

High: Further research is very unlikely to change our confidence in the estimate of effect; **Moderate:** Further research is likely to have an important impact on our confidence in the estimate of effect and may change the estimate**.**

Low: Further research is very likely to have an important impact on our confidence in the estimate of effect and will likely change the estimate. **Very low:** Any estimate of effect is very uncertain.

**CI,** Confidence Interval; **CMVR,** cytomegalovirus retinitis; **DFE,** Dilated fundus exam; **GRADE**, Grading of Recommendations, Assessment, Development and Evaluation; **PLHIV,** People Living with HIV.

### Strengths

In LMICs, patients with CMVR often need to travel long distances to access eye care services, and delays in referral and diagnosis can lead to progression of retinal damage, irreversible vision loss, and a significant impact on visual function and overall quality of life [21]. In this context, integrating teleretinal screening into primary HIV care facilities may play a strategic role in facilitating earlier detection and timely referral.

This is the first systematic review and meta-analysis focused on the diagnostic accuracy of teleretinal screening for CMVR among PLHIV. We employed a comprehensive and inclusive search strategy, without restrictions on age, CD4 count, language, or publication date, to ensure that potentially relevant studies were included. Two reviewers independently conducted data extraction and evaluated the risk of bias, helping to ensure objectivity and reduce potential bias.

Given that most individual studies on this topic included only a limited number of participants, conducting a systematic review with meta-analysis was essential to strengthen the evidence-based data. By pooling data from multiple studies, this review achieved a sufficiently large sample size, allowing for more reliable estimates of diagnostic accuracy and broader applicability of the findings.

### Limitations

The limited number of included studies, with some subgroup analyses based on single studies, restricts the robustness of pooled estimates and limits the strength of inference, warranting cautious interpretation of the findings; however, this also reflects the current lack of research in this area, emphasizing the relevance and need for this systematic review to synthesize the available evidence.

All included studies reported outcomes at the eye-level, and patient-level data were available only explicitly in one study. This may introduce unit-of-analysis bias due to within-patient correlation and could potentially overestimate diagnostic accuracy. However, eye-level analysis remains clinically relevant in the context of CMVR, as diagnosis, disease monitoring, and management decisions, including the administration of intravitreal ganciclovir, are performed on a per-eye basis. Each eye may present with differing disease involvement, and therefore, evaluating diagnostic performance at the eye-level reflects real-world clinical practice.

The exclusion of ungradable images in the included studies may have inflated estimates of diagnostic accuracy and limited the applicability to real-world screening settings; however, restricting the analysis to evaluable eyes, as determined by DFE, was necessary to minimize misclassification bias due to poor-quality images and to ensure valid comparison with the reference standard.

#### Quality of included studies.

Overall, the methodological quality of included studies was generally acceptable, with most domains demonstrating low risk of bias. However, concerns were identified in the patient selection domain, where unclear reporting of recruitment or enrollment methods may introduce selection bias. One study included previously diagnosed CMVR cases, which may have overestimated diagnostic accuracy, while others excluded younger populations, limiting applicability to broader PLHIV groups. In contrast, the index test and reference standard domains were consistently assessed as low risk of bias, with DFE as the gold standard. The flow and timing domain was also judged to be low risk, reflecting appropriate intervals between testing and inclusion of evaluable eyes to minimize misclassification.

**Generalizability.** Almost all studies were conducted in Asia and in tertiary healthcare settings, which may limit the generalizability of the findings to broader clinical settings. While U-MICs fall within the broader LMIC classification, no studies from LICs or L-MICs were identified, somehow limiting applicability to more resource-constrained settings. Nevertheless, the broader implementation potential may be informed by existing teleretinal screening programs in other regions. Teleretinal screening has been successfully used for conditions such as diabetic retinopathy across a range of LMIC settings and has even been integrated with artificial intelligence, demonstrating its feasibility in primary care and community-based settings [28]. A study by Du et al. (2024) also explored the use of deep learning systems for screening CMVR in these settings [29]. These established models provide a practical framework that could be leveraged for CMVR screening among PLHIV with advanced HIV disease, rather than requiring the development of entirely new systems. Integration into existing teleretinal screening infrastructure may enhance access to early detection and referral, particularly in settings with limited specialist availability, although adaptation to local context, including workforce training, imaging capacity, and referral systems, remains essential. Thus, additional research in primary care or community-based settings is recommended, as this review aims to integrate teleretinal screening into point-of-care settings, such as primary HIV care facilities.

### Applicability of findings to the review question

In CMVR, peripheral lesions are often missed during initial screening, which can progress toward the posterior pole, increasing the risk of visual loss if left untreated. Regular follow-up screenings are crucial for detecting these advancing lesions once they come into the viewable range of the fundus camera [21]. In this review, we were unable to include studies that conducted multiple screenings for PLHIV based on timely follow-ups. The median progression rate of CMVR toward the fovea in ganciclovir-untreated patients was approximately 24 μm/day, indicating that the disease generally advances slowly [30]. This slow progression provides a window of opportunity for detection before the macula becomes threatened, leading to visual loss. In line with this, Shah et al. (2013) reported that a three-month screening interval was adequate to identify CMVR lesions before they reached the macula or the optic disc, which are key events leading to significant visual loss [22]. Taken together, the slow radial spread of CMVR and the evidence supporting a three-month monitoring schedule explain why a three-month screening interval is sufficient and appropriate. Accounting for the three-month screening interval could have improved diagnostic accuracy in teleretinal screening for CMVR.

## Conclusion

Teleretinal screening for CMVR among PLHIV demonstrates consistently high specificity and potentially useful sensitivity; however, substantial uncertainty remains due to imprecision in sensitivity estimates and a limited evidence base, as reflected by the low certainty of evidence for sensitivity using the GRADE framework. These findings suggest that teleretinal screening may help support early detection and timely referral, particularly among PLHIV.

In this context, teleretinal screening enables trained technicians to perform retinal imaging across a range of clinical settings, thereby reducing the burden on ophthalmologists for initial evaluations. When combined with clear enrollment criteria, screening programs can prioritize PLHIV at high risk, allowing for a more efficient use of specialist resources.

### Implications for practice

Teleretinal screening may be considered as part of a targeted screening strategy for CMVR among PLHIV with advanced HIV disease, particularly those with CD4 counts <100 cells/mm^3^ and most critically <50 cells/mm^3^, where disease burden and diagnostic yield are highest. This approach may support earlier detection and referral, especially in settings with limited access to ophthalmologists. Integration into primary care systems should be approached cautiously and may be best achieved by leveraging existing teleretinal screening platforms, such as those used in diabetic retinopathy screening programs.

In future CMVR screening applications, this study can provide additional clinical criteria, particularly CD4 count, to remote graders, which may help improve diagnostic sensitivity and specificity.

### Implications for research

Further high-quality studies are needed to reduce uncertainty in diagnostic accuracy estimates, particularly in LMICs. Future research may consider patient-level analyses and evaluate real-world screening conditions, including broader population representation and diverse healthcare settings beyond tertiary centers.

From a policy perspective, these findings may inform global health strategies, including those led by the WHO and UNAIDS, in exploring the potential role of teleretinal screening within HIV care. However, implementation will require a clearer understanding of CMVR burden across settings. Future research should also investigate the development and validation of affordable and portable retinal imaging technologies, including mobile-based and artificial intelligence–assisted systems. Particular attention should be given to devices capable of capturing peripheral retinal lesions, which remain technically challenging but essential for accurate CMVR detection‌‌.

## Supporting information

S1 Table Search strategy. (PDF)

S1 Checklist PRISMA-DTA checklist. (PDF)

S1 Appendix Appendix‌‌. (PDF)
