# Supplementary material for: Diagnostic test accuracy of teleretinal screening for cytomegalovirus retinitis among people living with HIV: A systematic review and meta-analysis
Source: PLOS Glob Public Health. 2026 May 22;6(5):e0006327. doi: 10.1371/journal.pgph.0006327 (PMC13196956; doi:10.1371/journal.pgph.0006327)
Supplement: S1 Table — (PDF) [file pgph.0006327.s001.pdf]

All searches were conducted without any language or publication year restrictions on March 1, 2025.

1. Medical Literature Analysis and Retrieval System Online (MEDLINE) via PubMed

Table 1. Search strategy in our study on the electronic database, PubMed.

**Target Condition**		
#1	All Fields	Cytomegalovirus Retinitis OR CMVR
**Intervention**		
#2	All Fields	Teleretinal Screening OR Teleophthalmology OR Telemedicine OR Screening
**Outcome**		
#3	All Fields	Diagnostic Accuracy OR Sensitivity OR Specificity
#4	#1 AND #2 AND #3	

2. Cochrane Central Register of Controlled Trials (CENTRAL)

Table 2. Search strategy in our study on the electronic database, CENTRAL.

**Target Condition**		
#1	MeSH	Cytomegalovirus retinitis OR CMVR
**Intervention**		
#2	MeSH	Teleretinal Screening OR Teleophthalmology OR Telemedicine OR Screening
**Outcome**		
#3	MeSH	Diagnostic Accuracy OR Sensitivity OR Specificity
#4	#1 AND #2 AND #3	

3. Cumulative Index to Nursing and Allied Health Literature (CINAHL) through EBSCOhost

Table 3. Search strategy in our study on the electronic database, CINAHL.

**Target Condition**		
#1	All Text	Cytomegalovirus retinitis OR CMVR
**Intervention**		
#2	All Text	Teleretinal Screening OR Teleophthalmology OR Telemedicine OR Screening
**Outcome**		
#3	All Text	Diagnostic Accuracy OR Sensitivity OR Specificity
#1 AND #2 AND #3		

4. Scopus

Table 4. Search strategy in our study on the electronic database, Scopus.

**Target Condition**		
#1	All Fields	Cytomegalovirus retinitis OR CMVR
**Intervention**		
#2	All Fields	Teleretinal Screening OR Teleophthalmology OR Telemedicine OR Screening
**Outcome**		
#3	All Fields	Diagnostic Accuracy OR Sensitivity OR Specificity
#1 AND #2 AND #3		

5. Web of Science

Table 5. Search strategy in our study on the electronic database, Web of Science.

**Target Condition**		
#1	All Fields	Cytomegalovirus retinitis OR CMVR
**Intervention**		
#2	All Fields	Teleretinal Screening OR Teleophthalmology OR Telemedicine OR Screening
**Outcome**		
#3	All Fields	Diagnostic Accuracy OR Sensitivity OR Specificity
#1 AND #2 AND #3		
