# Supplementary material for: Diagnostic test accuracy of teleretinal screening for cytomegalovirus retinitis among people living with HIV: A systematic review and meta-analysis
Source: PLOS Glob Public Health. 2026 May 22;6(5):e0006327. doi: 10.1371/journal.pgph.0006327 (PMC13196956; doi:10.1371/journal.pgph.0006327)
Supplement: S1 Appendix — (PDF) [file pgph.0006327.s003.pdf]

S1 Appendix

Table A: Studies Assessed in Full-Text
Table B: Risk of Bias Assessment (QUADAS-2)
Table C: Diagnostic Accuracy Data of Included Studies

A total of 593 records were retrieved from CENTRAL, MEDLINE via PubMed, CINAHL, Scopus, and Web of Science. Following deduplication, 560 articles underwent title and abstract screening, of which 546 were excluded. Fourteen studies were subsequently assessed in full text for eligibility based on the predefined criteria.

Table A. Studies assessed in full text for eligibility against the predefined criteria.

Study Title	Author/s, year	Reason for Inclusion or Exclusion
Study to evaluate efficacy of midriatic-digital-images when screening retinal-complications in people living with HIV	Adrianzén et al., 2022	Excluded: Wrong Target Condition
Accuracy and reliability of telemedicine for diagnosis of cytomegalovirus retinitis	Ausayakhun et al., 2011	**Included**
Screening indices for cytomegalovirus retinitis in patients with human immunodeficiency virus	Butler and Friedman, 1992	Excluded: Wrong Study Design
Comparison of autophotomontage software programs in eyes with CMV retinitis	Chen et al., 2011	Excluded: Wrong Population
Deep learning system for screening AIDS-related cytomegalovirus retinitis with ultra-wide-field fundus images	Du et al., 2024	Excluded: Wrong Reference Standard
Utility of ultra-wide-field imaging for screening of AIDS-related cytomegalovirus retinitis	Du et al., 2020	**Included**
Telemedicine screening for cytomegalovirus retinitis at the point of care for human immunodeficiency virus infection	Jirawison et al., 2015	**Included**
Automated cytomegalovirus retinitis screening in fundus images	Kingkosol et al., 2020	Excluded: Wrong Study Design
Telemedicine diagnosis of eye disorders by direct ophthalmoscopy	Marcus et al., 1998	Excluded: Wrong Index Test
Screening for cytomegalovirus retinitis in HIV-positive and AIDS patients	Sandy et al., 1995	Excluded: Wrong Index Test
A Cytomegalovirus retinitis screening program: Evaluation of enrollment criteria for HIV patients in Singapore	Shah et al., 2014	Excluded: Wrong population
Telemedicine screening for cytomegalovirus retinitis using digital fundus photography	Shah et al., 2013	**Included**
Cytomegalovirus retinitis screening using machine learning technology	Srisuriyajan et al., 2022	**Included**
Telemedicine diagnosis of cytomegalovirus retinitis by nonophthalmologists	Yen et al., 2014	Excluded: Wrong Reference Standard

Table B. Risk of bias assessment of included studies using QUADAS-2 tool.

Domain/Signaling Questions	Ausayakhun 2011	Du 2020	Jirawison 2015	Shah 2013	Srisuriyajan 2022
**1. PATIENT SELECTION**					
**1A.1** Was a consecutive or random sample of patients enrolled?	Unclear	Unclear	Unclear	Unclear	Unclear
**1A.2** Was a case-control design avoided?	Yes	Yes	Yes	Yes	Yes
**1A.3** Did the study avoid inappropriate exclusions?	No	Yes	No	Yes	Yes
**1A.4 Risk of bias:** Could the selection of patients have introduced bias?	High	Unclear	Unclear	Unclear	Unclear
**1B Concerns regarding applicability:** Were there concerns that the included patients do not match the review question?	None	None	Yes	Yes	Yes
**2. INDEX TEST**					
**2A.1** Were the index test results interpreted without knowledge of the results of the reference standard?	Yes	Yes	Yes	Yes	Yes
**2A.2** If a threshold (or operating point) was used, was it pre-specified?	Not specified	Not specified	Not specified	Not specified	Not specified
**2A.4 Risk of bias:** Could the conduct or interpretation of the index test have introduced bias?	No	No	No	No	No
**2B. Concerns regarding applicability:** Were there concerns that the index test, its conduct, or interpretation differ from the review question?	None	None	None	None	None

Domain	Ausayakhun 2011	Du 2020	Jirawison 2015	Shah 2013	Srisuriyajan 2022
**3. REFERENCE STANDARD**					
**3A.1** Is the reference standard likely to classify the target condition correctly?	Yes	Yes	Yes	Yes	Yes
**3A.2** Was the reference standard result interpreted without knowledge of the result of the index test?	Yes	Yes	Yes	Yes	Yes
**3A.3 Risk of bias:** could the reference standard, its conduct, or its interpretation have introduced bias?	No	No	No	No	No
**3B. Concerns regarding applicability:** Were there concerns that the target condition, as defined by the reference standard, does not match the review question?	None	None	None	None	None
**4. FLOW AND TIMING**					
**4A.1** Was there an appropriate interval between index test(s) and reference standard?	Yes	Yes	Yes	Yes	Yes
**4A.2** Did all patients receive a reference standard?	Yes	Yes	Yes	Yes	Yes
**4A.3** Did all patients receive the same reference standard?	Yes	Yes	Yes	Yes	Yes
**4A.4** Were all patients included in the analysis?	Yes; discrepancy explained	Yes	Yes	Yes; discrepancy explained	Yes; discrepancy explained
**4A.5 Risk of bias:** Could the patient flow have introduced bias?	No	No	No	No	No

Table C. Diagnostic accuracy data of included studies

Study	Total No. of Eyes	TP	FP	FN	TN	Sensitivity	Specificity
Ausayakhun 2011	182	77	16	12	77	86.52	82.80
Du 2020	186	26	0	1	159	96.30	100
Jirawison 2015	205	6	2	15	182	28.57	98.91
Shah 2013	724	23	26	0	675	100	96.29
Srisuriyajan 2022	163	21	6	11	125	65.63	95.42

FN, false negative; **FP**, false positive; **TN**, true negative; **TP**, true positive.

REFERENCE

1. Adrianzén R, Rioja M, Manrique A, García Tello AV. Study to evaluate efficacy of midriatic-digital-images when screening retinal-complications in people living with HIV. *International journal of STD & AIDS*. 2022;33(8):773-776. doi: https://doi.org/10.1177/09564624221100941

2. Ausayakhun S, Skalet AH, Jirawison C, et al. Accuracy and reliability of telemedicine for diagnosis of cytomegalovirus retinitis. *American Journal of Ophthalmology*. 2011;152(6):1053-1058.e1. doi: https://doi.org/10.1016/j.ajo.2011.05.030

3. Butler GA, Friedman AH. Screening indices for cytomegalovirus retinitis in patients with human immunodeficiency virus. *The Mount Sinai journal of medicine, New York*. 1992;59(1):61-65. https://pubmed.ncbi.nlm.nih.gov/1310346/

4. Chen J, Ausayakhun S, Ausayakhun S, et al. Comparison of autophotomontage software programs in eyes with CMV retinitis. *Investigative Opthalmology & Visual Science*. 2011;52(13):9339. doi: https://doi.org/10.1167/iovs.11-8322

5. Du KF, Chen C, Huang XJ, et al. Utility of ultra-wide-field imaging for screening of AIDS-related cytomegalovirus retinitis. *Ophthalmologica Journal international d'ophtalmologie International journal of ophthalmology Zeitschrift fur Augenheilkunde*. 2020;244(4):334-338. doi: https://doi.org/10.1159/000512634
6. Du K, Dong L, Zhang K, et al. Deep learning system for screening AIDS-related cytomegalovirus retinitis with ultra-wide-field fundus images. *Heliyon*. 2024;10(10):e30881. doi: https://doi.org/10.1016/j.heliyon.2024.e30881

7. Jirawison C, Yen M, Leenasirimakul P, et al. Telemedicine screening for cytomegalovirus retinitis at the point of care for human immunodeficiency virus infection. *JAMA Ophthalmology*. 2015;133(2):198. doi: https://doi.org/10.1001/jamaophthalmol.2014.4766

8. Kingkosol P, Pooprasert P, Choopong P, Hunchangsith B, Laksanaphuk V, Tantibundhit C. Automated cytomegalovirus retinitis screening in fundus images. *Annual International Conference of the IEEE Engineering in Medicine and Biology Society IEEE Engineering in Medicine and Biology Society Annual International Conference*. 2020;2020:1996-2002. doi: https://doi.org/10.1109/EMBC44109.2020.9175461

9. Marcus DM, Brooks SE, Ulrich LD, et al. Telemedicine diagnosis of eye disorders by direct ophthalmoscopy. *Ophthalmology*. 1998;105(10):1907-1914. doi: https://doi.org/10.1016/s0161-6420(98)91040-5

10. Sandy C, Ferris J, Bloom P, Coker R, Pinching A, Migdal C. Screening for cytomegalovirus retinitis in HIV-positive and AIDS patients. *QJM : monthly journal of the Association of Physicians*. 1995;88(12):899-903. https://pubmed.ncbi.nlm.nih.gov/8593550/

11. Shah JM, Leo SW, Lee TL, et al. A Cytomegalovirus retinitis screening program: Evaluation of enrollment criteria for HIV patients in Singapore. *Ocular Immunology and Inflammation*. 2014;23(5):362-370. doi: https://doi.org/10.3109/09273948.2014.902472

12. Shah JM, Leo SW, Pan JC, et al. Telemedicine screening for cytomegalovirus retinitis using digital fundus photography. *Telemedicine and e-Health*. 2013;19(8):627-631. doi: https://doi.org/10.1089/tmj.2012.0233

13. Srisuriyajan P, Cheewaruangroj N, Polpinit P, Laovirojjanakul W. Cytomegalovirus retinitis screening using machine learning technology. *Retina*. 2022;Publish Ahead of Print. doi: https://doi.org/10.1097/iae.0000000000003506

14. Yen M, Ausayakhun S, Chen J, et al. Telemedicine diagnosis of cytomegalovirus retinitis by nonophthalmologists. *JAMA Ophthalmology*. 2014;132(9):1052. doi: https://doi.org/10.1001/jamaophthalmol.2014.1108
